# Orexin A alleviates neuroinflammation via OXR2/CaMKKβ/AMPK signaling pathway after ICH in mice

**DOI:** 10.1186/s12974-020-01841-1

**Published:** 2020-06-15

**Authors:** Tao Li, Weilin Xu, Jinsong Ouyang, Xiaoyang Lu, Prativa Sherchan, Cameron Lenahan, Giselle Irio, John H. Zhang, Jianhua Zhao, Yongfa Zhang, Jiping Tang

**Affiliations:** 1 Department of Neurosurgery, The First People’s Hospital of Yunnan Province (Kunhua Hospital/The Affiliated Hospital of Kunming University of Science and Technology), Yunnan, 650032 China; 2 Department of Physiology and Pharmacology, School of Medicine, Loma Linda University, 11041 Campus St, Loma Linda, CA 92354 USA; 3 Department of Neurosurgery, The Second Affiliated Hospital of Zhejiang University School of Medicine, Zhejiang, 310009 Hangzhou China; 4 Burrell College of Osteopathic Medicine, 3501 Arrowhead Dr, Las Cruces, NM 88001 USA

**Keywords:** Orexins, Orexin receptors, Intracerebral hemorrhage, Secondary brain injury, Neuroinflammation

## Abstract

**Background:**

Orexins are two neuropeptides (orexin A, OXA; orexin B, OXB) secreted mainly from the lateral hypothalamus, which exert a wide range of physiological effects by activating two types of receptors (orexin receptor 1, OXR1; orexin receptor 2, OXR2). OXA has equal affinity for OXR1 and OXR2, whereas OXB binds preferentially to OXR2. OXA rapidly crosses the blood-brain barrier by simple diffusion. Many studies have reported OXA’s protective effect on neurological diseases via regulating inflammatory response which is also a fundamental pathological process in intracerebral hemorrhage (ICH). However, neuroprotective mechanisms of OXA have not been explored in ICH.

**Methods:**

ICH models were established using stereotactic injection of autologous arterial blood into the right basal ganglia of male CD-1 mice. Exogenous OXA was administered intranasally; CaMKKβ inhibitor (STO-609), OXR1 antagonist (SB-334867), and OXR2 antagonist (JNJ-10397049) were administered intraperitoneally. Neurobehavioral tests, hematoma volume, and brain water content were evaluated after ICH. Western blot and ELISA were utilized to evaluate downstream mechanisms.

**Results:**

OXA, OXR1, and OXR2 were expressed moderately in microglia and astrocytes and abundantly in neurons. Expression of OXA decreased whereas OXR1 and OXR2 increased after ICH. OXA treatment significantly improved not only short-term but also long-term neurofunctional outcomes and reduced brain edema in ipsilateral hemisphere. OXA administration upregulated p-CaMKKβ, p-AMPK, and anti-inflammatory cytokines while downregulated p-NFκB and pro-inflammatory cytokines after ICH; this effect was reversed by STO-609 or JNJ-10397049 but not SB-334867.

**Conclusions:**

OXA improved neurofunctional outcomes and mitigated brain edema after ICH, possibly through alleviating neuroinflammation via OXR2/CaMKKβ/AMPK pathway.

## Introduction

Orexins (OXs) are not one protein, but two peptides secreted mainly by specific neurons in the lateral hypothalamus [1, 2]. Orexins or orexin peptides consist of two neuropeptides: orexin A (OXA) and orexin B (OXB) [3], both of which act broadly on central and peripheral nervous system to regulate feeding, sleep cycle, metabolism, neuroendocrine, and immune activities [4, 5]. Orexin receptors (OXRs) are also comprised of two types: orexin receptor 1 (OXR1) and orexin receptor 2 (OXR2), both belong to G protein-coupled receptors (GPCRs) family [3]. It has been confirmed by numerous studies that OXA has approximately equal affinity for OXR1 and OXR2, whereas OXB preferentially binds to OXR2 [1, 3]. Kastin et al. have reported that OXA can cross the blood-brain barrier (BBB) rapidly by means of simple diffusion but failed to observe the same with OXB [6]. Although distributed extensively on cell membrane in brain tissue, the two receptors are not expressed evenly but with an obvious preference for different brain regions. OXR1 tends to localize in tenia tecta, dorsal raphe nucleus, and cornu ammonis, while OXR2 is expressed predominantly in basal ganglia [7, 8]. A number of studies have reported that OXs/OXRs involve regulating multiple pathological processes, particularly in neurological diseases. Harada et al. reported that the nerve injury was improved with the intracerebroventricular (i.c.v.) injection of OXA in a cerebral ischemia model in mice [9]. Similar neuronal protection was observed in a cerebral ischemia model in rats [10]. Xiong et al. found that OXA treatment could relieve inflammation after cerebral ischemia in mice by downregulating the mRNA expression of TNFα and IL-6 [11].

Hemorrhagic stroke accounts for 20–30% of all strokes [12]. Though less than one third, its more acute onset, more fatal course, and higher mortality and morbidity make it a radical challenge for clinical treatment [12, 13]. Intracerebral hemorrhage (ICH) is defined as non-traumatic, non-tumor caused hemorrhage in the brain tissue, and it contributes to 60–75% of hemorrhagic stroke [12, 14]. Conventional treatment strategies for ICH emphasize the importance of early removal of hematoma using surgical procedures. However, after decades of practice with more and more advanced surgical techniques, the prognosis of ICH has not improved. In recent years, a hypothesis aiming to explain the intractable brain injury by ICH has been put forward. The brain injury caused by ICH can be generalized as primary brain injury (PBI) and secondary brain injury (SBI) [15, 16]. The former is the direct damage by hematoma; the latter consists in a series of pathologies and pathological process such as toxicity of hematoma metabolites, oxidative stress, inflammation, and neuronal apoptosis [16–19]. The SBI is one of the major reasons for the poor outcome of ICH, and thus treatment strategies should re-focus on how to prevent SBI from progressing.

We hypothesized that OXA could exert a neuroprotective effect on SBI after ICH by alleviating neuroinflammation. Therefore, the model of ICH was established, and multiple studies were performed to confirm our hypothesis and to explore the mechanisms.

## Methods

### Animals

A total of 180 adult male CD-1 mice used in the study (35 ± 5 g, 56 ± 5 days old) were purchased from Charles River Laboratories (Wilmington, MA, USA) and housed in a specialized animal room with 12 h light/dark cycles and strictly controlled temperature (25 °C) and humidity (50–70%), and were allowed to obtain food and water freely. All the procedures on animals were approved by the Institutional Animal Care and Use Committee at Loma Linda University and conformed to the National Institutes of Health Guide for the Care and Use of Laboratory Animals.

### ICH model establishment

The ICH model was established using a stereotactic injection system to inject the autologous arterial blood into the right basal ganglion [20]. In brief, the CD-1 mouse was anesthetized with a mixture (20 ml/kg, *i.p.*) of ketamine (100 mg/kg) and xylazine (10 mg/kg). An incision was made on the scalp to expose the skull and the bregma. The mouse was put in a prone position, and its head was fixed on the stereotactic frame; eyes were protected with an artificial tear ointment (Rugby, Livonia, MI). Taking the bregma as the origin, a burr hole was drilled on the right side following the coordinates (medial-lateral 2.2 mm, anterior-posterior 0.2 mm). The burr hole was sealed with bone wax temporarily, and the mouse was unfixed from the fame. An incision was incised on right groin to expose the femoral artery, and then the distal of the artery was ligated. The proximal blood flow was discontinued using a clip, and a minor incision was performed on the femoral artery using micro-scissors under a surgical microscope. The catheter (MRE025, Micro-Renathane) was inserted through the minor incision to draw arterial blood after releasing the clip. A volume of 50 μl blood was obtained. The proximal was ligated, and the incision on the right groin was closed up temporarily using medical tape. A volume of 40 μl blood was drawn using a micro-syringe (Hamilton 80500, NV, USA). The mouse head was re-fixed on the frame, and the needle of the syringe was inserted through the burr hole at the depth of 3.5 mm. The injection was initiated with the pump system (Stoelting, Harvard Apparatus, Holliston, MA) at a rate of 3 μl/min, for duration of 10 min, and a total volume of 30 μl. To prevent a backflow, the depth could be reduced 0.5 mm every 2 min. The syringe was kept in place for another 10 min when the injection was complete. Then, the syringe was retreated slowly; the burr hole was sealed with bone wax, and the incisions on the scalp and the groin were sutured. The sham mice received identical procedures including blood draw and micro-syringe insertion, except for a real injection.

### Drug administration

Recombinant OXA peptide (O6012, Sigma-Aldrich, St. Louis, MO, USA) was dissolved in saline to form the solutions with the concentration of 200 ng/μl (high dose), 60 ng/μl (medium dose), and 20 ng/μl (low dose), respectively [10]. The OXA drug solution was administered intranasally (*i.n*.) [21] 1 h after ICH induction, with 2.5 μl in each nostril (5.0 μl per mouse). The same volume (5.0 μl) of saline was administered in the vehicle group of OXA. CaMKKβ inhibitor, STO-609 (S8274, Selleck Chem, Houston, TX, USA), was dissolved in 5% dimethyl sulfoxide (DMSO) to yield a drug solution with a concentration of 5 mg/ml, and 100 μl of the drug solution was injected intraperitoneally (*i.p.*) 1 h after ICH induction [22]. SB-334867 (S7585, Selleck Chem, Houston, TX, USA) [23, 24] or JNJ-10397049 (sc-491320, Santa Cruz, Dallas, TX, USA) [25], which is respectively OXR1 or OXR2 selective antagonist, was dissolve in 5% DMSO to form the drug solution (10 mg/ml), and then 100 μl was injected (*i.p.*) 1 h after ICH induction. The vehicle group received the same volume (100 μl) of 5% DMSO.

### Neurobehavioral tests

The modified Garcia test, forelimb placement test, and corner turn test were performed to evaluate the short-term (24 h and 72 h) neurological outcomes. Foot fault test, rotarod test, and Morris water maze were employed to evaluate the mid- and long-term (1–4 weeks) neurofunction after ICH. The foot fault test and the rotarod test were both performed on the day before ICH induction (baseline) and then performed after every 7 days for consecutive 3 weeks after ICH. Details of the tests were as previously described [26–29].

*Modified Garcia test* includes seven individual trials, which assess the spontaneous activity, symmetry of limb movement, forelimb stretching, climbing, proprioception, response to vibrissae stroke, and lateral turn. Each trial is scored 0 to 3, where 0 is the worst and 3 is the best. The total score (0–21) was obtained by adding up the scores of seven trials to evaluate the neurological function.

*Forelimb placement test* was performed by holding the trunk of the mouse and stroking its left vibrissae along the edge of a platform. The result was interpreted as the percentage of the times of left forelimb placement on the platform to the total times of vibrissae strokes.

*Corner turn test* was conducted using an instrument which were two boards forming an angle of 30° vertically on the platform. The mouse was put on the platform and led to make a turn in the corner. The result was the percentage of the acts of turning by the acts of left turning.

*Foot fault test* required a mesh board (100 cm × 20 cm; CleverSys Inc., VA, USA) which hung horizontally held at the two ends. The mouse was placed on one end and induced to walk along the mesh board to another end. A recording device was set below the mesh board to record the walk and the steps into the mesh (missteps).

*Rotarod test* was performed using an apparatus (Columbus Instruments, Columbus, OH). The mice to be tested were placed in each lane on the rotating cylinder at a speed of 5 revolutions per minute (RPM). The falling latency which is defined as the time duration when a mouse stabilizes himself on the rotating cylinder without falling was recorded.

*Morris water maze test* required a compilation of devices including a circular pool (diameter of 150 cm, depth of 50 cm), a platform (diameter of 15 cm), and a tracking system with analyzing software (Noldus Ethovision; Noldus Information Technology, Wageningen, The Netherlands). Before the test, warm water (25 °C) was pumped in the pool to reach a height of 30 cm and dyed black with non-toxic ink. The test was performed at 21 days after ICH and continued for 6 days. On the first test day (D 1), the platform was placed in one quadrant and 3 cm above the water surface for the mice to reach and stay. Then, the platform was placed in other 3 quadrants in a clockwise or counterclockwise order to repeat the tests. From the second test day (D 2) and on, the platform was placed 2 cm under the water surface in the same order as D 1 to repeat the tests. On the last test day (D 6), the platform was removed, and the probe test was performed to generate a trail heatmap. All the tests were carried out in a dark room, and each mouse was tested 10 times with a 10-min break.

### Hematoma volume

The mouse was euthanized and perfused transcardially with phosphate buffered saline (PBS, 0.01 M, pH 7.40, 4 °C), and then its right hemisphere was dissected and added with the same PBS (1500 μl) to be homogenized thoroughly into a suspension. After the centrifugation (12,000 RPM, 30 min, 4 °C), the supernatant (100 μl) was extracted and mixed with the Drabkin’s reagent (400 μl, Sigma-Aldrich, MO, USA) to incubate 15 min in a dark room. Using a spectrophotometer (Thermo Scientific^TM^ GENESYS 10S, USA) to detect the absorbance (540 nm), the hemoglobin concentration and hematoma volume of each sample could be calculated by comparing the absorbance with a standard curve [30, 31].

### Brain water content

The euthanized mouse had the brain dissected into ipsilateral basal ganglion (Ipsi-BG), ipsilateral cortex (Ipsi-CX), contralateral basal ganglion (Con-BG), contralateral cortex (Con-CX), and cerebellum (Cerebel) rapidly. All the parts acquired from one brain were weighed for wet weight (WW) respectively in a certain order which should also be followed when weighting others. After that, the samples were transferred into an oven to be dried (105 °C, 48 h). The ratio of DW to WW was calculated, and the brain water content (BWC) was achieved by subtracting the ratio from 100% [32].

### Western blot

Western blot (WB) analysis was performed as previously described [33]. Briefly, the right hemisphere was homogenized and centrifuged 12,000 RPM, 30 min, 4 °C, and the supernatant was standardized into a solution with the equivalent protein concentration. The protein solution was mixed with a loading buffer reagent and then denatured (95 °C, 10 min). The subsequent protein samples (10 μl) were loaded into the wells of the polyacrylamide gel to have the electrophoresis. After protein separation, the gel was covered with a PVDF membrane for protein transfer. When the transfer was complete, the PVDF membrane was rinsed with Tris buffered saline with Tween 20 (TBST, pH 7.40) and then soaked in a non-fat milk solution (5%) for 1 h. The primary antibodies were diluted per manufacturer’s instruction with a non-fat milk solution (5%) and then co-incubated (12 h, 4 °C). After the incubation, the membranes were washed with TBST and co-incubated (2 h, 25 °C). The probed membranes were washed with TBST and further processed with an ECL reagent before exposed on photographic films. Relative density of greyscale of protein bands was calculated using the ImageJ software (National Institutes of Health, MD, USA). Primary antibodies included anti-OXA (1:2000; PA5-70436, Thermo Fisher, MA, USA), anti-OXR1 (1:1000; PA5-77566, Thermo Fisher, MA, USA), anti-OXR2 (1:1000; PA5-77567, Thermo Fisher, MA, USA), anti-p-CaMKKβ (1:1000; #12818, Cell Signaling Technology, Inc., MA, USA), anti-CaMKKβ (1:1000; #16810, Cell Signaling Technology, Inc., MA, USA), anti-p-AMPK (1:1000; #5759, Cell Signaling Technology, Inc., MA, USA), anti-AMPK (1:1000; #5831, Cell Signaling Technology, Inc., MA, USA), anti-p-NFκB (1:1000; #8242, Cell Signaling Technology, Inc., MA, USA), anti-IL-1β (1:1000; #63124, Cell Signaling Technology, Inc., MA, USA), anti-TNFα (1:1000; #11948, Cell Signaling Technology, Inc., MA, USA), and anti-β-actin (1:5000; ab8226, Abcam, MA, USA). Secondary antibodies included anti-rabbit (1:5000; ab205718, Abcam, MA, USA) and anti-mouse (1:5000; ab205719, Abcam, MA, USA).

### Enzyme linked immunosorbent assay

Using enzyme linked immunosorbent assay (ELISA), inflammation-related cytokines including IL-1β, TNFα, IL-6, IL-12, IL-4, and IL-10 were detected and quantified 24 h after ICH [34]. The manufacturer’s protocol was followed as provided in the ELISA kit. In brief, brain tissue extracted, and protein supernatant was acquired as described above. The protein sample or standard was mixed with antibody cocktail in pre-coated 96-well microplate and then incubated at 25 °C for 1 h. Next, the wells were washed using wash buffer three times. The development solution was added to each well to co-incubate in a microplate, and stop solution was added, and the microplate was analyzed using a spectrophotometer to calculate optical density (OD) at 450 nm. By comparing the OD of target protein with standard curve, the protein concentration was quantified. The following ELISA kits were used: IL-1β (ab197742, Abcam, MA, USA), TNFα (ab208348, Abcam, MA, USA), IL-6 (ab222503, Abcam, MA, USA), IL-12 (ab236717, Abcam, MA, USA), IL-4 (ab221833, Abcam, MA, USA), and IL-10 (ab255729, Abcam, MA, USA).

### Immunofluorescence staining

The immunofluorescence (IF) staining was performed as previously described [35, 36]. In brief, mice were euthanized and perfused with PBS (0.01 M, pH 7.40, 4 °C) transcardially first and then perfused with 4% paraformaldehyde (PFA) solution (pH 7.40, 4 °C). The cerebrums were dissected before soaked in the same PFA solution for fixation (48 h, 4 °C). The samples were transferred to a 30% sucrose solution for dehydration (72 h, 4 °C). After that, the brain samples were imbedded and frozen (− 80 °C) and then sliced into coronal slices (8-μm thick) which were mounted on glass slides. Before staining, the slides were washed with PBS and processed with a mixture of Triton X-100 (0.3%) and donkey serum (5%) for 1 h. Next, the slides were incubated with primary antibodies overnight (12 h, 4 °C). Then, the corresponding secondary antibodies were added and allowed to co-incubate (1 h, 25 °C). Afterwards, the slides were washed and sealed with a DAPI reagent (Thermo Fisher, MA, USA) and subsequently examined under a fluorescence microscope (Leica, Germany). Primary antibodies used were anti-OXA (1:100; ab120212, Abcam, MA, USA), anti-OXR1 (1:100; PA5-77566, Thermo Fisher, MA, USA), anti-OXR2 (1:100; PA5-77567, Thermo Fisher, MA, USA), anti-NeuN (1:100; ab177487, Abcam, MA, USA), anti-Iba-1 (1:100; ab178847, Abcam, MA, USA), and anti-GFAP (1:100; ab7260, Abcam, MA, USA). Secondary antibodies used were anti-rabbit (1:100; ab150077, Abcam, MA, USA) and anti-mouse (1:100; ab150117, Abcam, MA, USA).

### Study design

The animal groups and number of mice used in the study are listed in Supplementary Material Table S1.

#### Experiment 1: time-course of OXA, OXR1, and OXR2 (*n* = 38)

Thirty-six mice were randomly allotted to 6 groups: sham (*n* = 6), ICH 3 h (*n* = 6), ICH 6 h (*n* = 6), ICH 12 h (*n* = 6), ICH 24 h (*n* = 6), and ICH 72 h (*n* = 6). The protein expression of OXA, OXR1, and OXR2 in brain tissue was quantified using WB analysis. Two mice were used to study the expression and localization of OXA, OXR1, and OXR2 in neurons, microglia, and astrocytes at 24 h after ICH by performing the IF staining (*n* = 2).

#### Experiment 2: evaluate protective effects of OXA (*n* = 72)

Thirty mice were randomly assigned into 5 groups: sham (*n* = 6), ICH + vehicle (*n* = 6), ICH + OXA (20 ng/μl) (*n* = 6), ICH + OXA (60 ng/μl) (*n* = 6), and ICH + OXA (200 ng/μl) (*n* = 6). Modified Garcia test, forelimb placement test, and corner turn test were performed at 24 h to explore whether OXA could improve the neurofunction and what the best dosage was. After the tests, mice brains were harvested for hematoma volume quantification. Next, 18 mice were randomly assigned into 3 groups: sham (*n* = 6), ICH + vehicle (*n* = 6), and ICH + OXA (optimal dose) (*n* = 6) to repeat the aforementioned 3 neurobehavioral tests at 72 h and then to calculate the brain water content. Additionally, 24 mice were randomly assigned into 3 groups: sham (*n* = 8), ICH + vehicle (*n* = 8), ICH + OXA (optimal dose) (*n* = 8) to evaluate the mid- and long-term neurological outcomes.

#### Experiment 3: mechanisms of the protective effect of OXA (*n* = 42)

Forty-two mice were randomly assigned into 7 groups: sham (*n* = 6), ICH + vehicle (*n* = 6), ICH + OXA (optimal dose) (*n* = 6), ICH + OXA + DMSO (*n* = 6), ICH + OXA + STO-609 (*n* = 6), ICH + OXA + SB-334867 (*n* = 6), and ICH + OXA + JNJ-10397049 (*n* = 6) which were used in the following experiments. First, we performed western blot using the 3 groups: sham (*n* = 6), ICH + vehicle (*n* = 6), and ICH + OXA (optimal dose) (*n* = 6) to analyze the protein expression of OXA, OXR1, and OXR2. Second, we gathered 5 groups: sham (*n* = 6), ICH + vehicle (*n* = 6), ICH + OXA (optimal dose) (*n* = 6), ICH + OXA + DMSO (*n* = 6), and ICH + OXA + STO-609 (*n* = 6) to detect the protein expression of p-CaMKKβ/CaMKKβ, p-AMPK/AMPK, p-NFκB, IL-1β, and TNFα to confirm the signaling pathway. Next, we gathered 5 groups: sham (*n* = 6), ICH + vehicle (*n* = 6), ICH + OXA (optimal dose) (*n* = 6), ICH + OXA + SB-334867 (*n* = 6), and ICH + OXA + JNJ-10397049 (*n* = 6) to study the responses when OXR1 or OXR2 was selectively inhibited, for the purpose of revealing the roles that OXR1 and OXR2 played in the signaling pathway. Finally, ELISA was performed to quantify inflammation-related cytokines to further confirm western blot results in 6 groups: sham (*n* = 6), ICH + vehicle (*n* = 6), ICH + OXA (*n* = 6), ICH + OXA + STO-609 (*n* = 6), ICH + OXA + SB-334867 (*n* = 6), and ICH + OXA + JNJ-10397049 (*n* = 6).

### Statistical analysis

All data were tested for distribution patterns. Analysis of variance (ANOVA) was used for the data in normal distribution; Kruskal-Wallis test with the Bonferroni correction was used for the data in abnormal distribution. Statistical results are presented as mean ± standard deviation (SD) and considered significant when the *p* value was less than 0.05.

## Results

### Mortality rate

No mice in the sham group (0/40) died, while the mortality was 7.1% (10/140) after ICH induction. However, no significant differences were noted in mortalities between the ICH groups.

### Time-course expression and localization of OXA, OXR1, and OXR2

**Fig. 1:**
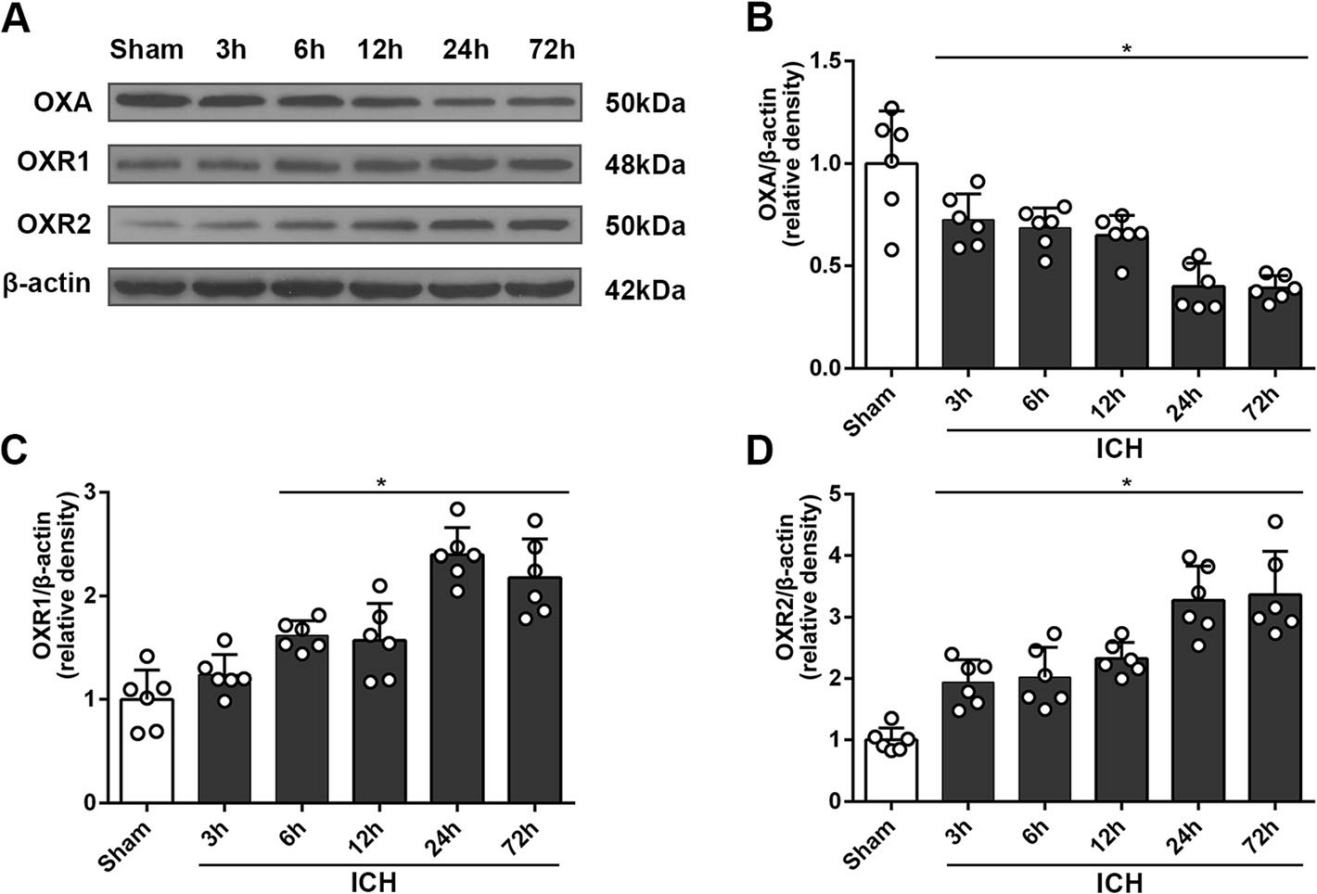
Time-course study of OXA, OXR1, and OXR2 using western blot. **a** Representative protein bands. **b** Western blot quantitative analyses of OXA, one-way ANOVA, *p < 0.05 vs. sham group. Error bars represent the means ± SD, *n* = 6 per group. **c** Western blot quantitative analyses of OXR1, one-way ANOVA, *p < 0.05 vs. sham group. Error bars represent the means ± SD, *n* = 6 per group. **d** Western blot quantitative analyses of OXR2, one-way ANOVA, *p < 0.05 vs. sham group. Error bars represent mean ± SD, *n* = 6 per group

**Fig. 2:**
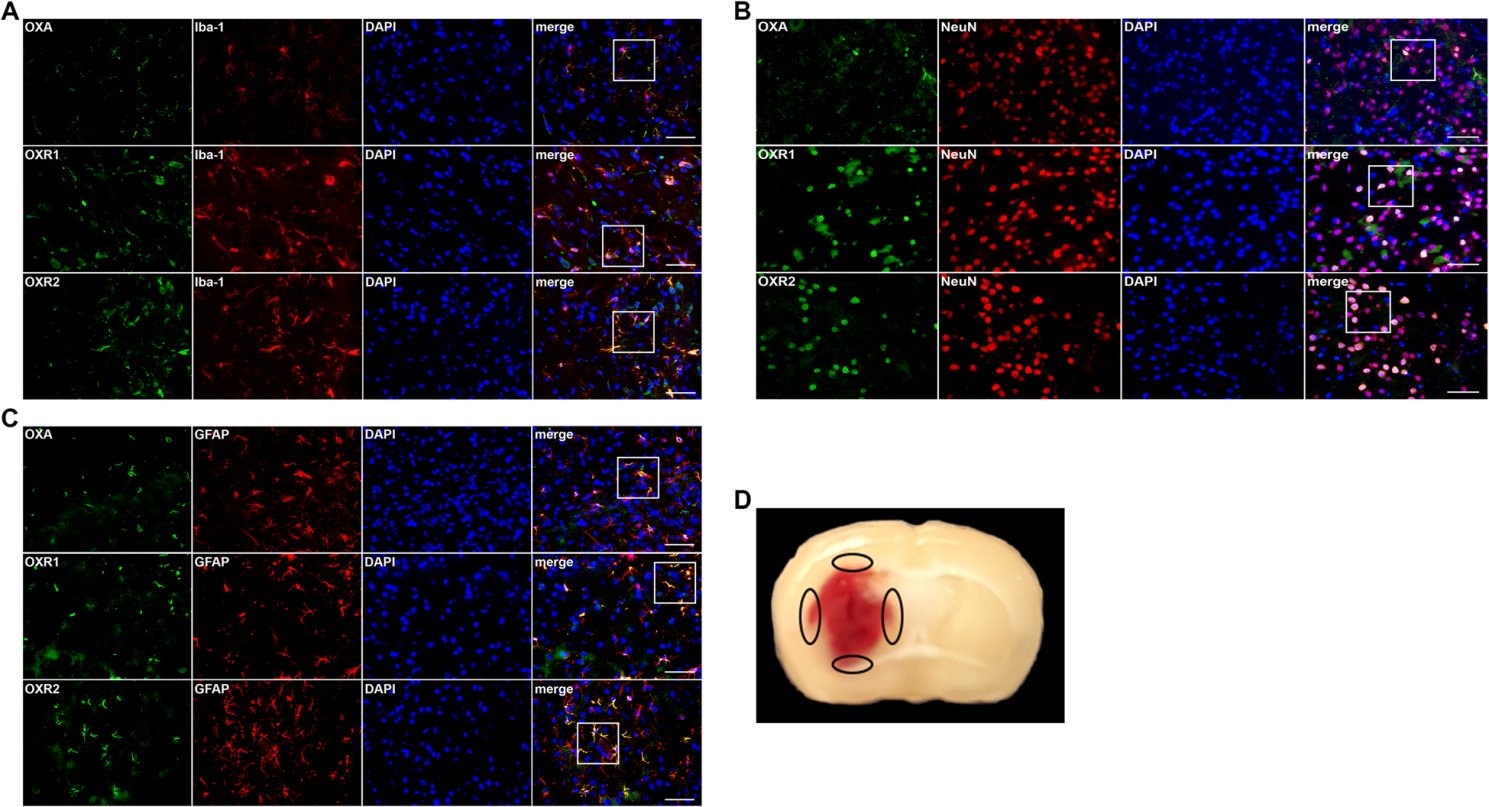
Immunofluorescence staining of mice brain sections at 24 h after ICH. **a** Representative pictures of co-staining of OXA/OXR1/OXR2 (green) with Iba-1 (red, microglia). **b** Representative pictures of co-staining of OXA/OXR1/OXR2 (green) with NeuN (red, neurons). **c** Representative pictures of co-staining of OXA/OXR1/OXR2 (green) with GFAP (red, astrocytes). Scale bars = 50 μm, *n* = 2. **d** A typical mouse brain section 24 h after ICH. The black circles in the peri-hematomal region indicate the brain areas which were observed under the fluorescence microscope to obtain the representative immunofluorescence pictures

Western blot results suggested that the expression of OXA decreased significantly at 3 h after ICH and continued decreasing until 24 h (*p <* 0.05, Fig. 1a, b), whereas that of OXR1 and OXR2 increased and peaked at 24 h (Fig. 1a, c, d). Immunofluorescence (IF) staining results showed that OXA, OXR1, and OXR2 were expressed moderately in microglia and astrocytes, and abundantly in neurons (*p* < 0.05, Fig. 2).

### Short-term neurological outcomes, hematoma volume, and brain water content after ICH

**Fig. 3:**
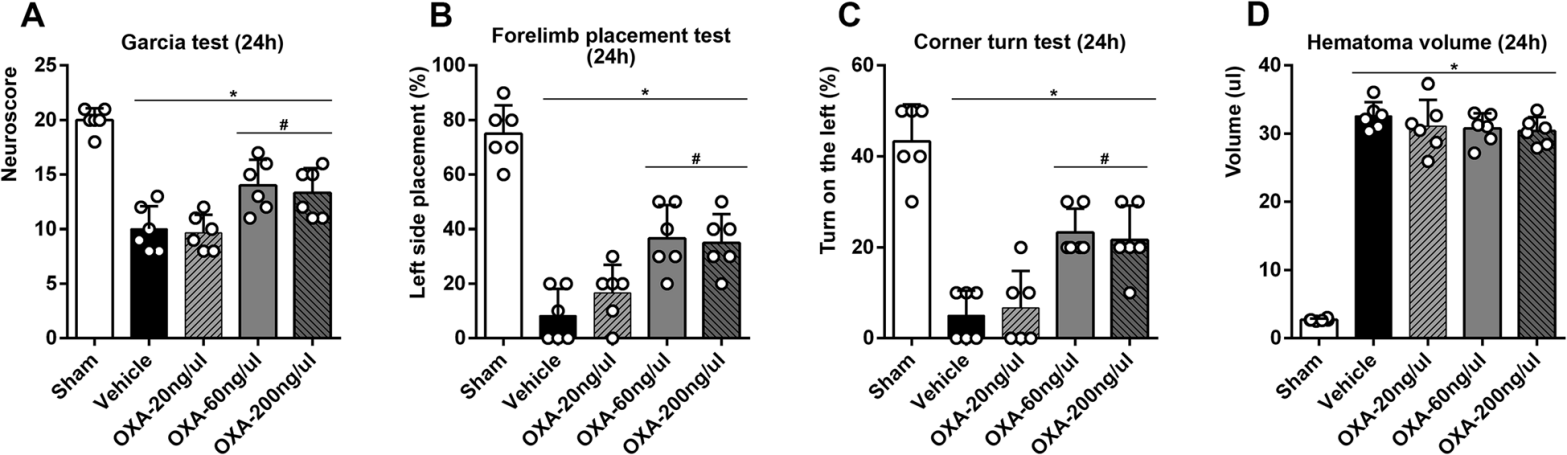
Short-term outcome studies at 24 h after ICH. **a** Garcia test, **b** forelimb placement test, and **c** corner turn test at 24 h all showed that the neurological function were significantly decreased after ICH. Low dose (20 ng/μl) OXA administration failed to change the results; medium (60 ng/μl) and high dose (200 ng/μl) OXA treatment significantly improved the neurological outcomes; however, no significance was observed between the two groups. Kruskal-Wallis test with the Bonferroni correction, *p < 0.05 vs. sham group, #*p* < 0.05 vs. vehicle group. Error bars represent the means ± SD, *n* = 6 per group. **d** Hematoma volume was quantitatively estimated. It was significantly elevated after ICH; however, there was no significant differences between all the groups. Kruskal-Wallis test with the Bonferroni correction, *p < 0.05 vs. sham group. Error bars represent mean ± SD, *n* = 6 per group

Garcia test, forelimb placement test, and corner turn test at 24 h after ICH consistently showed that neurofunctions were significantly damaged (*p* < 0.05, Fig. 3a–c). Low dose (20 ng/μl) OXA treatment failed to reverse the neurological deficits, while medium and high dose OXA significantly improved the neurofunctions (*p* < 0.05, Fig. 3a–c). However, no significant differences were observed in terms of the protective effect between the two dosages. The hematoma volume was also evaluated at 24 h after ICH, and no significant differences were confirmed, which suggested that OXA treatment could not change the hematoma volume (*p* < 0.05, Fig. 3d).

**Fig. 4:**
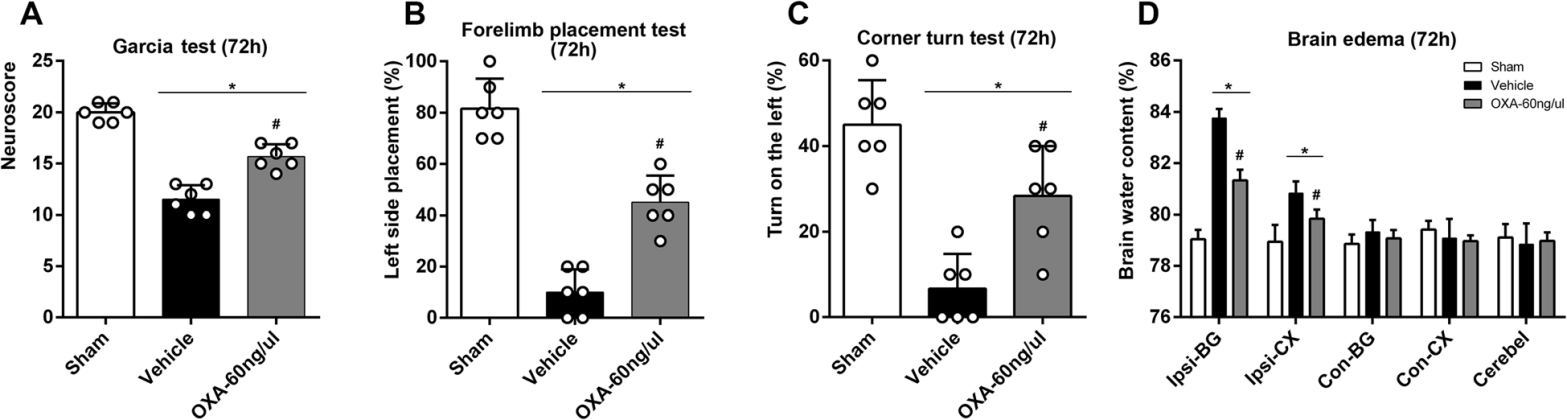
Short-term outcome studies at 72 h after ICH. **a** Garcia test, **b** forelimb placement test, and **c** corner turn test at 72 h showed that neurological function was significantly impaired after ICH, and OXA treatment significantly improved outcomes. Kruskal-Wallis test with the Bonferroni correction, *p < 0.05 vs. sham group, #*p* < 0.05 vs. vehicle group. Error bars represent the means ± SD, *n* = 6 per group. **d** Brain water content was evaluated 72 h after ICH. There was no significant difference in brain edema in the contralateral brain hemisphere or the cerebellum. Brain edema increased in the ipsilateral hemisphere observed after ICH, which was significantly ameliorated with OXA administration. One-way ANOVA, *p < 0.05 vs. sham group, #*p* < 0.05 vs. vehicle group. Error bars represent mean ± SD, *n* = 6 per group. BG, basal ganglia; CX, cortex; Cerebel, cerebellum

Based on the test results at 24 h, the medium dose (60 ng/μl) was adopted as the optimal dosage for the following tests. Garcia test, forelimb placement test, and corner turn test were re-performed at 72 h after ICH. Similarly, the neurofunctions endured significant deficits after ICH induction, while the mice that received medium dose OXA had significantly improved neurofunctions (*p* < 0.05, Fig. 4a–c). In the meantime, the brain water content was measured to quantify brain edema. Although no significant differences on contralateral cerebral hemisphere and cerebellum were noted, brain water content in ipsilateral (right) basal ganglia and cortexes exhibited significant differences, which suggested the brain hemisphere on ICH induction side would have severe edema, and OXA administration effectively reduced the brain edema (*p* < 0.05, Fig. 4d).

### Mid- and long-term neurological outcomes after ICH

**Fig. 5:**
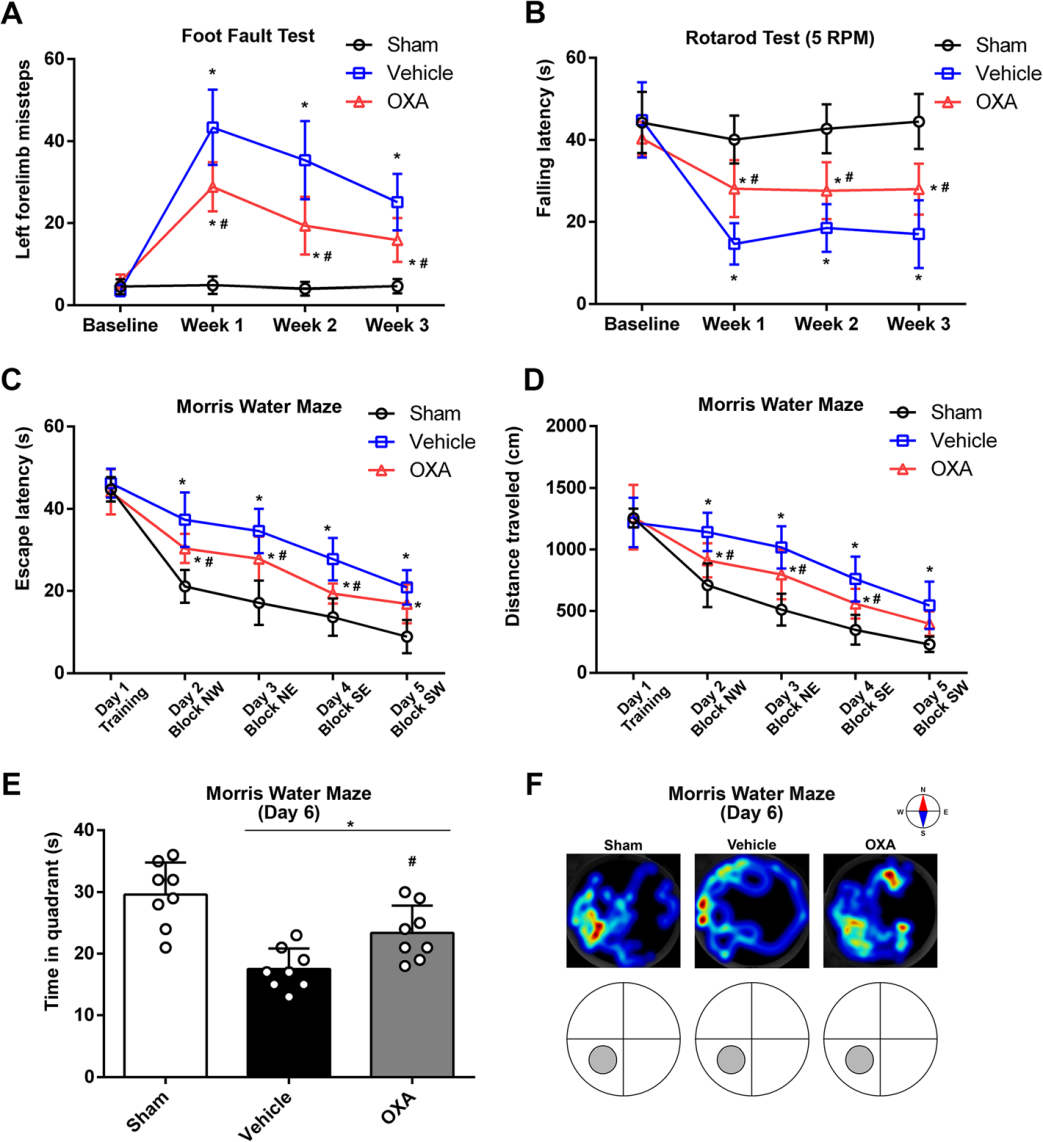
Mid- and long-term neurological outcomes after ICH. **a** Foot fault test and **b** rotarod test were performed before ICH (baseline) and 1 week, 2 weeks, and 3 weeks after ICH induction. There was no significant difference at the baseline, indicating the neurofunctions were not statistically divergent among the mice. After ICH induction, the neurofunctions were significantly impaired and were improved by OXA treatment. Two-way ANOVA, *p < 0.05 vs. sham group, #*p* < 0.05 vs. vehicle group. Error bars represent mean ± SD, *n* = 8 per group. Morris water maze test evaluated **c** escape latency and **d** distance traveled which showed no significant at the baseline on day1 of testing, while a significant reduction was observed at test days 2, 3, 4, and 5. The administration of OXA significantly rescued the functions at test days 2, 3, and 4. Two-way ANOVA, *p < 0.05 vs. sham group, #*p* < 0.05 vs. vehicle group. Error bars represent mean ± SD, *n* = 8 per group. **e** Time spent in the probe quadrant was decreased after ICH, which was significantly improved with OXA treatment. One-way ANOVA, *p < 0.05 vs. sham group, #*p* < 0.05 vs. vehicle group. Error bars represent mean ± SD, *n* = 8 per group. **f** Heatmap of swimming trails could distinctively reflect the above results. The trails were chaotic after ICH and improved by OXA treatment

For evaluating mid-term neurological outcomes, foot fault test and rotarod test were performed on the day before ICH induction (baseline) and the first, second, and third week after ICH. The results of missteps showed no significant differences on baseline, suggesting the mice from the three groups had no significant variations of motor function. The number of missteps significantly surged after ICH induction at weeks 1, 2, and 3, and it was decreased significantly using OXA treatment (*p* < 0.05, Fig. 5a). Likewise, rotarod test showed that there was no difference of falling latency on baseline, whereas a significant decrease on weeks 1, 2, and 3 after ICH induction. Administration of OXA significantly improved the falling latency (*p* < 0.05, Fig. 5b).

As for long-term neurological outcomes, Morris water maze test was performed at 21 days after ICH. No significant difference of escape latency was found on test day 1. The escape latency was significantly increased after ICH and was improved on test days 2, 3, and 4 significantly with OXA administration (*p* < 0.05, Fig. 5c). Consistent results were observed in the outcome of distance traveled. No significant was observed on test day 1 while a significant reduction was observed after ICH. OXA treatment could significantly decrease the distance traveled before reaching the target (*p* < 0.05, Fig. 5d). On test day 6, the time duration in the probe quadrant was measured and found to decrease significantly with ICH induction and was reversed by OXA (*p* < 0.05, Fig. 5e). This was visualized with the heatmap which showed that the swim trail was chaotic after ICH induction but improved by using OXA (*p* < 0.05, Fig. 5f).

### Anti-inflammatory mechanism of OXA treatment

**Fig. 6:**
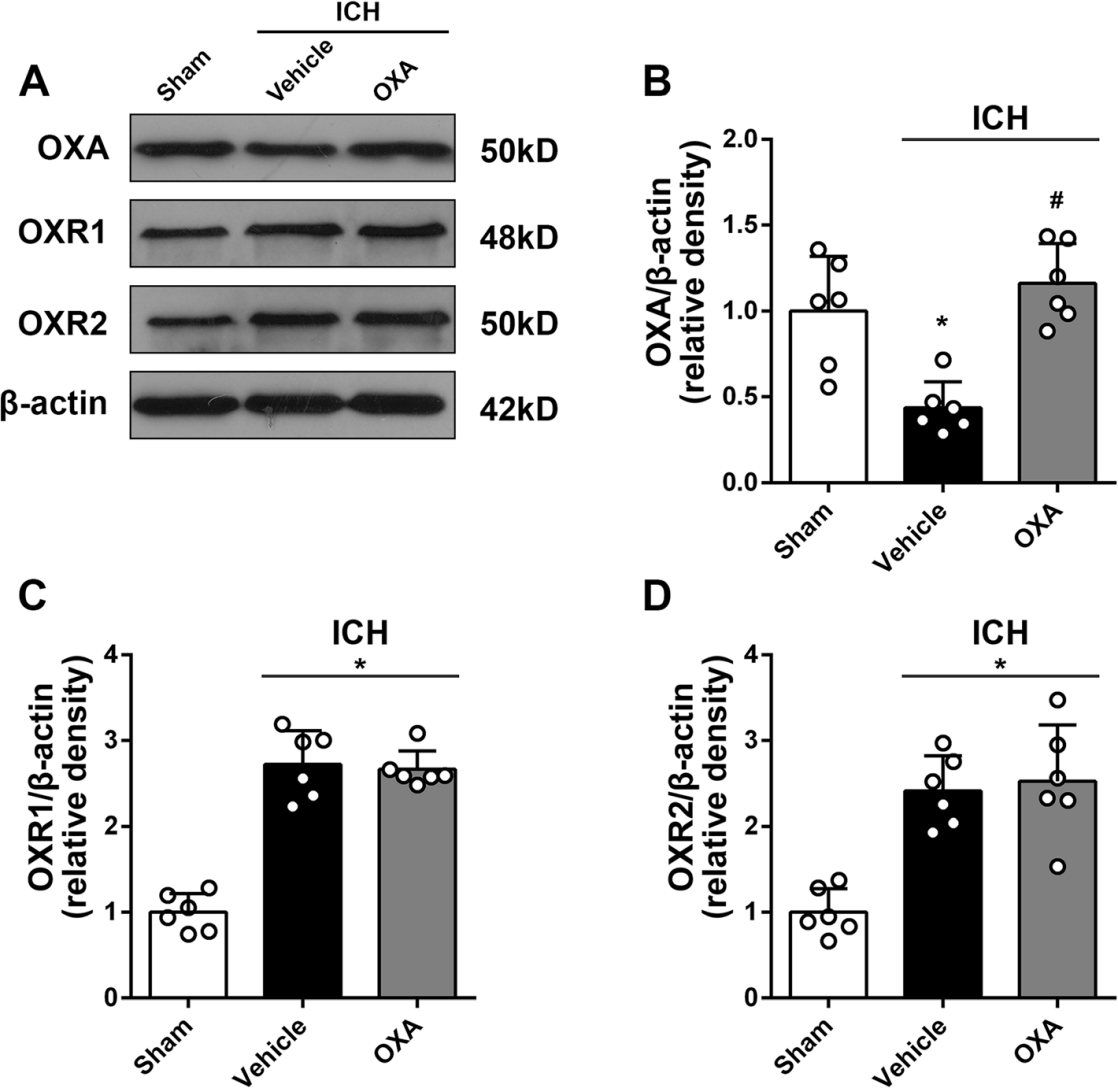
Western blot analysis of OXA and OXRs expression after intranasal OXA administration. **a** Representative protein bands and the quantitative analysis of **b** OXA, **c** OXR1, and **d** OXR2 at 24 h after ICH. OXA antibody could detect exogenous OXA protein in brain samples, which was significantly increased with OXA treatment. However, there was no significant change in OXR1 or OXR2 expressions after OXA treatment. One-way ANOVA, *p < 0.05 vs. sham group, #*p* < 0.05 vs. vehicle group. Error bars represent mean ± SD, *n* = 6 per group

**Fig. 7:**
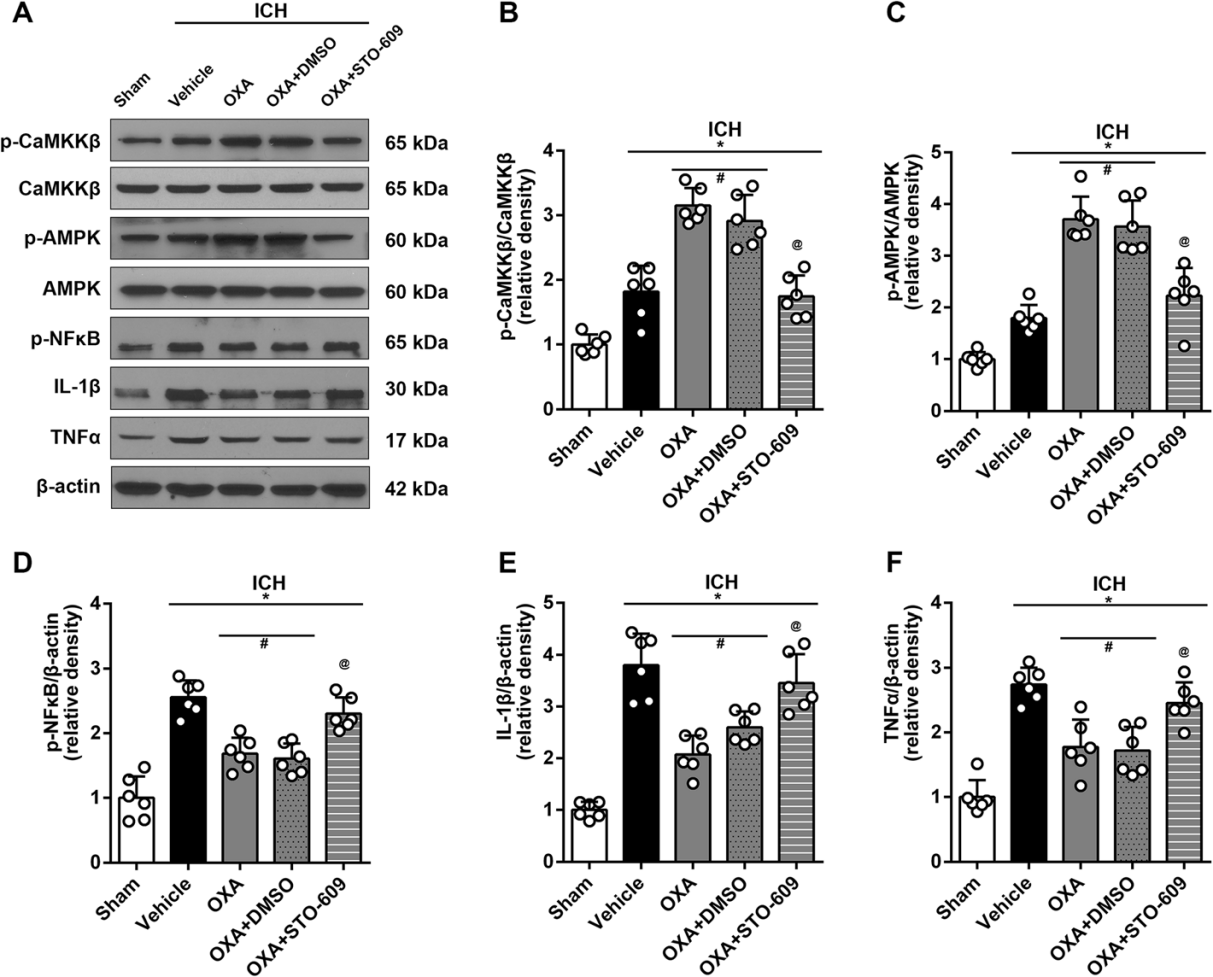
Western blot analysis showing anti-inflammatory effects of OXA. **a** Representative protein bands and the quantitative analysis of **b** ratio of p-CaMKKβ/CaMKKβ and **c** ratio of p-AMPK/AMPK at 24 h after ICH. The expression of p-CaMKKβ and p-AMPK was upregulated after ICH and was further elevated by OXA. This effect was reversed with STO-609 (p-CaMKKβ inhibitor). Consequently, the inflammatory factors including **d** p-NFκB, **e** IL-1β, and **f** TNFα were increased after ICH and were significantly suppressed by OXA. Consistently, this effect was reversed with STO-609. One-way ANOVA, *p < 0.05 vs. sham group, #*p* < 0.05 vs. vehicle group, @*p* < 0.05 vs. OXA group. Error bars represent mean ± SD, *n* = 6 per group

Western blot results showed that exogenous OXA was detected in the brain samples after ICH (Fig. 6a). The administration of OXA significantly increased OXA protein content in the brain tissue (*p* < 0.05, Fig. 6a, b). However, the expression of the two receptors OXR1 and OXR2 was not affected by OXA treatment (Fig. 6a, c, d). Moreover, OXA treatment significantly increased the expression of p-CaMKKβ (*p* < 0.05, Fig. 7a, b) and p-AMPK (*p* < 0.05, Fig. 7a, c), whereas it decreased the downstream inflammatory markers including p-NFκB, IL-1β, and TNFα (*p* < 0.05, Fig. 7a, d–f). This effect was reversed by using selective p-CaMKKβ inhibitor (STO-609) (*p* < 0.05, Fig. 7a–f).

### Differential effects of OXR1 and OXR2 receptors

**Fig. 8:**
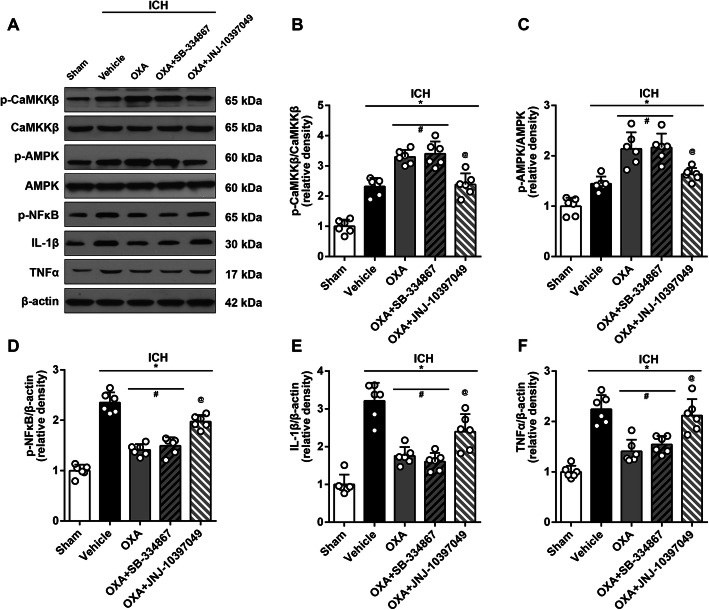
Western blot analysis evaluating the effects of OXR1 and OXR2. **a** Representative protein bands and the quantitative analysis of **b** ratio of p-CaMKKβ/CaMKKβ and **c** ratio of p-AMPK/AMPK at 24 h after ICH which was upregulated with OXA. The expressions of inflammatory factors **d** p-NFκB, **e** IL-1β, and **f** TNFα were significantly downregulated by OXA, which was consistent with the above results. Administration of SB-334867 (OXR1 antagonist) had no significant impact on the mice that received OXA, whereas JNJ-10397049 (OXR2 antagonist) significantly reversed the effects of OXA treatment. One-way ANOVA, *p < 0.05 vs. sham group, #*p* < 0.05 vs. vehicle group, @*p* < 0.05 vs. OXA group. Error bars represent mean ± SD, *n* = 6 per group

Consistently, OXA treatment significantly upregulated the expression of p-CaMKKβ (*p* < 0.05, Fig. 8a, b) and p-AMPK (*p* < 0.05, Fig. 8a, c) while downregulated the expressions of p-NFκB, IL-1β, and TNFα (*p* < 0.05, Fig. 8a, d–f). Administration of selective OXR1 inhibitor (SB-334867) did not change the expression of these proteins compared to those in OXA treatment group (*p* < 0.05, Fig. 8a–f). However, the mice that received selective OXR2 inhibitor (JNJ-10397049) significantly decreased the expression of p-CaMKKβ and p-AMPK while increasing expressions of p-NFκB, IL-1β, and TNFα (*p* < 0.05, Fig. 8a–f).

**Fig. 9:**
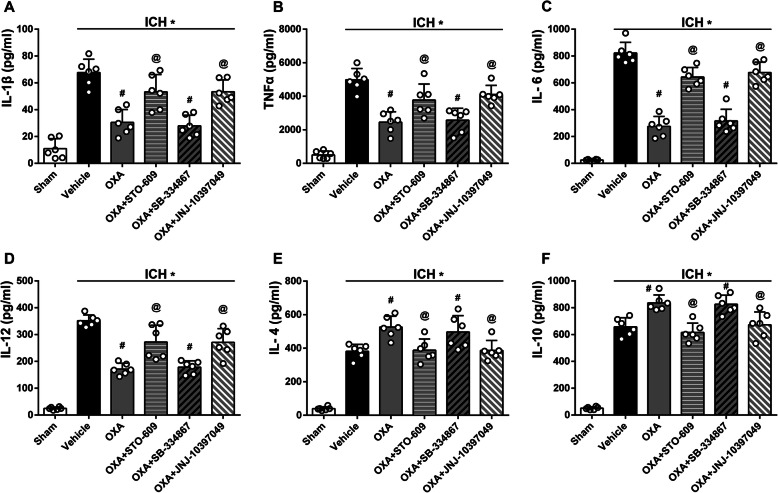
ELISA tests to evaluate inflammation-related cytokines with OXA treatment. The levels of pro-inflammatory cytokines including **a** IL-1β, **b** TNFα, **c** IL-6, and **d** IL-12 were significantly downregulated with OXA treatment, and the levels of anti-inflammatory cytokines including **e** IL-4 and **f** IL-10 were significantly upregulated with OXA treatment evaluated at 24 h after ICH. The effects of OXA were reversed by CaMKKβ inhibitor (STO-609) and OXR2 inhibitor (JNJ-10397049) but not OXR1 inhibitor (SB-334867). One-way ANOVA, *p < 0.05 vs. sham group, #*p* < 0.05 vs. vehicle group, @*p* < 0.05 vs. OXA group. Error bars represent mean ± SD, *n* = 6 per group

**Fig. 10:**
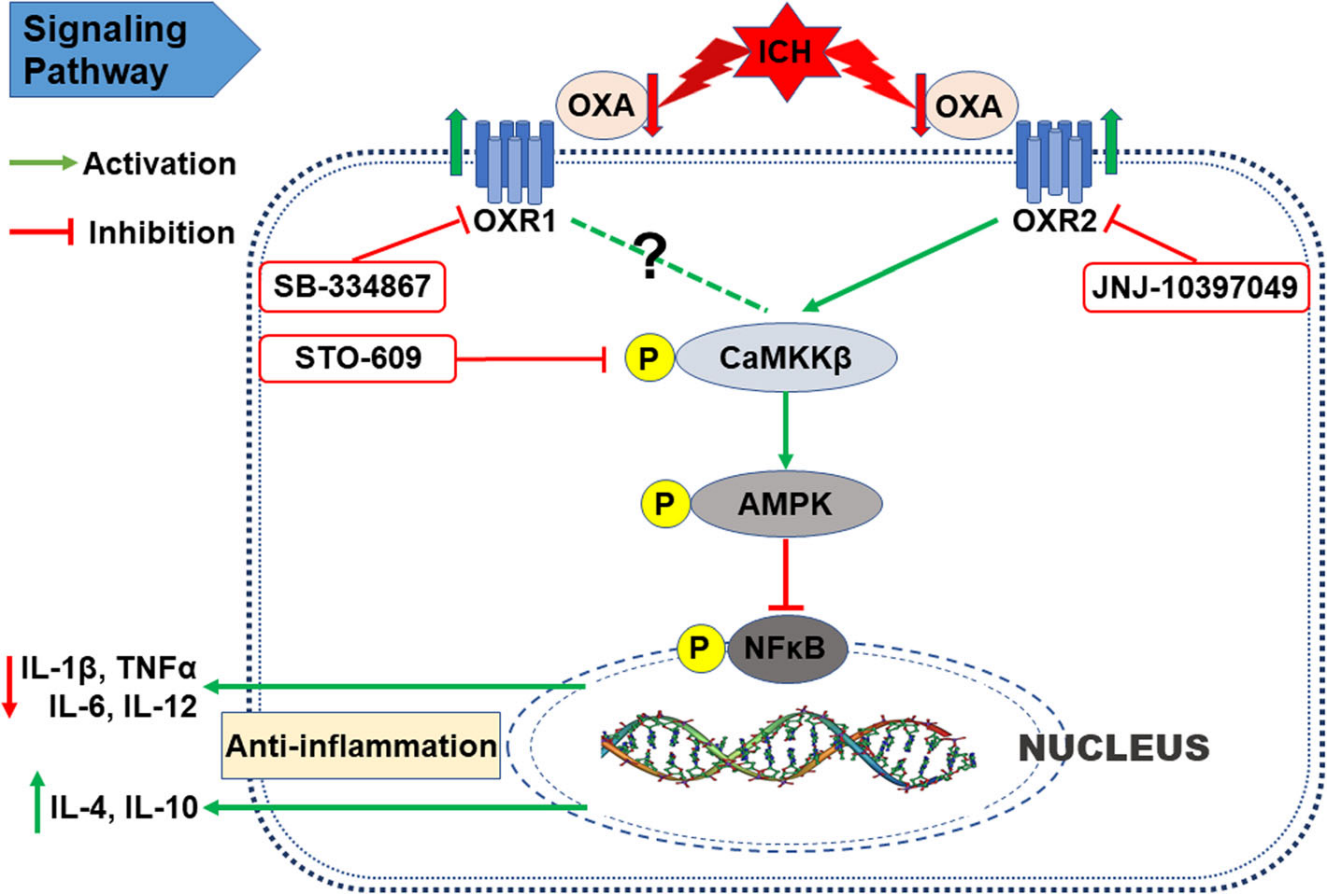
Anti-inflammatory signaling pathway mediated via OXA and OXR2 in ICH

Additionally, the anti-inflammatory mechanism of OXA and its receptors was evaluated using ELISA. The levels of pro-inflammatory cytokines including IL-1β, TNFα, IL-6, and IL-12 were significantly downregulated with OXA treatment (*p* < 0.05, Fig. 9a–d), whereas the levels of anti-inflammatory cytokines IL-4 and IL-10 were significantly upregulated with OXA treatment (*p* < 0.05, Fig. 9e, f). These effects of OXA were significantly reversed by CaMKKβ inhibitor (STO-609) and OXR2 inhibitor (JNJ-10397049) but not OXR1 inhibitor (SB-334867). These results suggest that the anti-inflammatory effects of OXA are mediated via OXR2 signaling pathway in ICH (Fig. 10).

## Discussion

In this study, we observed that the protein expression of OXA decreased while that of OXR1 and OXR2 increased significantly after ICH, and the three were expressed widely in microglia, astrocytes, and neurons. We also confirmed that exogenous OXA treatment significantly improved both the short-term and the mid- and long-term neurofunctions in experimental mice after ICH. The mechanism was dependent on the anti-inflammatory effect of OXA, and possibly through OXR2/CaMKKβ/AMPK signaling pathway.

Several clinical studies have highlighted that the level of OXs/OXRs is closely related to stroke incident. Dohi et al. reported that the level of OXA was significantly lower in the cerebrospinal fluid (CSF) in patients with subarachnoid hemorrhage (SAH) [37]. They also observed the same phenomenon in patients with ICH [38]. Ang et al. reported the consistent result that orexins level in the CSF decreased significantly and seemed inversely correlated to the severity of SAH [39]. Similarly, a persistent decline of OXA level in CSF was discovered in patients with ischemic stroke. Nakamachi et al. found that the protein expression of OXR1 was significantly induced after transient common carotid artery occlusion (tCCAO) in mice [40]. Although it is less studied and barely reported in stroke, OXR2 expression was demonstrated to be upregulated in other neurological diseases [41, 42] and cardiac diseases [43]. Our study results were consistent with these reports. We detected the protein expression of OXA, OXR1, and OXR2 at different time-points after ICH using western blot and confirmed that the level of OXA decreased whereas that of OXR1 and OXR2 increased significantly after ICH. In addition, the results from immunofluorescence staining showed that OXA, OXR1, and OXR2 were expressed widely in microglia and astrocytes, more abundantly in neurons. These study results suggested that the OXA/OXRs system was highly correlated with the pathologies of ICH and might be playing an important role. However, we did not evaluate the expression of OXA and OXRs at 72 h after ICH, and our study focused on brain injury at cellular and molecular level at the early stage (≤ 72 h) only. Also, future studies using cell culture and in vitro methods will be required to clarify the cell specific roles of OXA. Increasingly in recent years, the physiological properties of OXA/OXRs in neurological diseases have been verified. Some researchers claimed that OXs secreting neurons were reduced, and OXA seemed absent in specific brain regions of patients with narcolepsy, which suggested OXA was vitally necessary to maintain normal sleep and prevent narcolepsy [44, 45]. It was demonstrated that OXA had a neuroprotective effect; exogenous OXA injection (*i.c.v.*) efficiently diminished the infarction size and improve neurofunctions in a cerebral ischemia model in mice and rats [9, 10]. Hadadianpour et al. proved that the administration (*i.c.v.*) of OXA significantly ameliorated motor and cognitive functions of rats with Parkinson’s disease [46]. By using PET scan, Van de Bittner et al. found that intranasal administration of OXA achieved similar drug concentration and biological effect compared with intravenous route [21]. Based on these studies, we thus postulated that OXA should have similar neuroprotective effect on mice with ICH and evaluated the effects of intranasal OXA delivery to study its effect in ICH. By means of neurobehavioral tests including Garcia test, forelimb placement test, and corner turn test at 24 h and 72 h after ICH, together with brain edema quantification, we confirmed that OXA could significantly improve the short-term neurofunctions after ICH and ascertained the optimal dosage of OXA for the subsequent studies. The mid- and long-term neurofunctions were assessed using foot fault test, rotarod test, and Morris water maze test. We further confirmed that OXA was also effective in ameliorating the mid- and long-term neurofunctions after ICH.

In this study, we evaluated the effects of OXA and determined which of the orexin receptors were involved in protection after ICH. Several studies have reported that OXA has equal affinity for OXR1 and OXR2, while OXB has preference for OXR2 and can act on OXR1 with lower affinity [47, 48]. Additionally, OXA and OXB have similar molecular structure and biological effects. Given that OXA can bind to both orexin receptors, we chose to evaluate the effects of OXA since it would allow determining which of the two orexin receptors were involved in orexin mediated protection. Furthermore, in the mechanism study, we explored which of the two orexin receptors played a role in OXA mediated protection after ICH. Another rationale for not choosing OXB in this project was the study by Kastin et al. which reported that OXB cannot cross the BBB, but OXA can do so via a simple diffusion route [6]. The administration of OXB would require ICH mice to be subjected to additional invasive procedure such as intracranial injection. However, it will be important to study the effect of OXB in future studies. Furthermore, previous study by Van de Bittner et al. found that intranasal administration of OXA was able to achieve similar drug concentration and biological effect compared with intravenous route using PET scan study [21]. Also, the study by Kastin et al. demonstrated that OXA can cross the BBB [6]. Based on these studies, we chose to administer OXA via intranasal route. Furthermore, we observed that following intranasal administration, exogenous OXA was detected in the brain samples, and OXA content was significantly increased in brain tissue. However, the expression of OXR1 and OXR2 was not affected by OXA administration. We thus excluded the possibility that OXA exerts its effect by changing the protein expression of its receptors.

The over-activated inflammatory response in brain after ICH insult is considered a radical factor in SBI and to be responsible for the poor outcome [16, 49, 50]. Drugs or treatments with anti-inflammatory property in nervous system have often proven effective in alleviating SBI and improving neurological outcomes [51, 52]. It was reported that OXA pre-treatment could significantly downregulate the mRNA of TNFα and IL-6 in a model of cerebral ischemia, which meant the neuroinflammation was suppressed [11]. Many studies have demonstrated that the activated AMPK (p-AMPK) by CaMKKβ is a keystone regarding inflammation suppression in microglia [53, 54]. Wu et al. reported that AMPK could be activated by OXA via the L-type calcium channel [55]. Taken together, we deduced that OXA acted on its receptors to activate CaMKKβ, which in turn activated AMPK; the activated AMPK suppressed further activation of inflammatory factors. Results from western blot confirmed our deduction. The levels of p-NFκB, IL-1β, and TNFα were significantly increased after ICH, which meant a boost of inflammatory response. In the meantime, CaMKKβ and AMPK were also activated to limit the progression of the inflammation. OXA treatment significantly activated CaMKKβ and AMPK and downregulated the expression of p-NFκB, IL-1β, and TNFα. Moreover, the effect of OXA could be offset by STO-609, suggesting that the anti-inflammatory effect was dependent on CaMKKβ.

It has been noted that OXA/OXRs system plays versatile or even opposite effects in the same pathological process. In depression research, some researchers deem that OXA/OXRs system functions as a protector [56, 57], whereas some argue the opposite [58, 59]. Similar contradiction also exists in the research on Alzheimer’s disease [60, 61]. More commonly, the different roles that OXR1 and OXR2 might play have been reported. Scammell et al. reported that OXR2 or non-selective OXRs antagonists were effective in treating insomnia [62], suggesting OXR2 was more involved. Nishino et al. found that mutated OXR2 was related to narcolepsy in canine [63]. Price et al. proposed that OXR1 instead of OXR2 was more predominant in the regulation of dopamine signaling and cocaine self-administration [64]. Irving observed an increase of OXR1 mRNA but not OXR2 mRNA in rat model of brain ischemia [65]. In our study, we noted that the anti-inflammatory effect of OXA was significantly reversed by JNJ-10397049 (OXR2 inhibitor) while it seemed not to be affected by SB-334867 (OXR1 inhibitor). Thus, we believe the two types of receptors act differently; OXR2 is more involved in CaMKKβ/AMPK signaling pathway to mediate anti-inflammatory effect after ICH. The possible explanation, we speculate, is the receptor distribution and the downstream signaling pathway activation. It was reported that OXR2 has a higher expression density in basal ganglia, while OXR1 is more expressed in other brain regions such as tenia tecta, dorsal raphe nucleus, and cornu ammonis [7, 8]. A series of studies demonstrated that OXR1 and OXR2 could bind with other subunits to form different dimers to activate different signaling molecules downstream [66–69].

However, it is undeniable that our research has limitations. First, as the aforementioned, SBI includes multiple pathological processes; we investigated only inflammation after ICH and therefore are uncertain whether OXA/OXRs could alleviate SBI through other routes. Second, in terms of the inflammatory response after ICH, various signaling pathways participate. We have acknowledged the role of CaMKKβ/AMPK pathway but did not exclude the effect of other pathways. Third, though we have confirmed that OXR2 activated CaMKKβ by increasing its phosphorylation, the detail of the interaction remains unclarified. Fourth, we noted in our studies that OXR2 was more involved in the activation of CaMKKβ and anti-inflammatory effects after ICH. However, we have not explored the mechanism to explain why the two types of receptors act differently and whether OXR1 is negligible in regulating the inflammatory response after ICH. Lastly, we investigated neuroinflammation based on resident microglia and did not evaluate the role of peripheral immune cells which invade the injury site and may also be introduced from the injected blood. Resident and peripheral immune cells both contribute to neuroinflammation after ICH, and it will be critical to explore these mechanisms in future studies.

## Conclusions

Our study results sustain the conclusions that OXA can significantly improve neurofunctional outcomes and mitigate brain edema after ICH, possibly through the mechanism of alleviating neuroinflammation via OXR2/CaMKKβ/AMPK pathway. Recombinant OXA or chemical OXR2 agonists are currently available, thrift, and easy to administer to the patients with ICH, thus are promising drugs with a potential for use in clinical practice.

## Supplementary information

**Additional file 1:.** Table S1 Animal Groups and Number of Mice Used in the Study

## Data Availability

The datasets analyzed during the current study are available from the corresponding author on reasonable request.
