# Supplementary material for: Orexin A alleviates neuroinflammation via OXR2/CaMKKβ/AMPK signaling pathway after ICH in mice
Source: J Neuroinflammation. 2020 Jun 15;17:187. doi: 10.1186/s12974-020-01841-1 (PMC7294616; doi:10.1186/s12974-020-01841-1)
Supplement: Supplementary file 1 — Additional file 1:. Table S1 Animal Groups and Number of Mice Used in the Study [file 12974_2020_1841_MOESM1_ESM.docx]

| **Groups** | **Mortality** | **Excluded** |
| --- | --- | --- |
| **Experiment 1: Time course and IF** |  |  |
| Sham (n=8) | 0 (0/8) | 0 |
| ICH (3h, 6h, 12h, 24h, 72h) | 6.3% (2/32) | 2 |
| **Experiment 2.1: Short-term outcome study (24h and 72h)** |  |  |
| Sham (n=12) | 0 (0/12) | 0 |
| ICH+Vehicle (n=13) | 7.7% (1/13) | 1 |
| OXA-20ng/ul (n=6) | 0 (0/6) | 0 |
| *OXA-60ng/ul (n=13) | 7.7% (1/13) | 0 |
| OXA-200ng/ul (n=6) | 0 (0/6) | 0 |
| **Experiment 2.2: Long-term outcome study** |  |  |
| Sham (n=8) | 0 (0/8) | 0 |
| ICH+Vehicle (n=9) | 11.1% (1/9) | 0 |
| ICH+OXA (n=9) | 11.1% (1/9) | 1 |
| **Experiment 3: Mechanism study** |  |  |
| Sham (n=12) | 0 (0/12) | 0 |
| ICH+Vehicle (n=13) | 1 (1/13) | 0 |
| ICH+OXA (n=13) | 1 (1/13) | 0 |
| ICH+OXA+DMSO (n=7) | 14.3% (1/7) | 1 |
| ICH+OXA+STO-609 (n=6) | 0 (0/6) | 0 |
| ICH+OXA+SB-334867 (n=7) | 14.3% (1/7) | 0 |
| ICH+OXA+JNJ-10397049 (n=6) | 0 (0/6) | 0 |
| **TOTAL** |  |  |
| Sham | 0 (0/40) | 0 |
| ICH | 7.1% (10/140) | 5 |

**Table S1 Animal Groups and Number of Mice Used in the Study**

*OXA-60 ng/ul was the optimal dose and then selected for the following studies.

A total of 180 mice were used: 40 in sham group, 140 in ICH group. Additionally, 5 mice were excluded because of failure to establish the ICH model.

In **Experiment 3,** 20 mice were added for ELISA test: 6 in sham group, 7 in vehicle group and 7 in ICH + OXA group.
